# A pilot study investigating the efficacy of technology enhanced case based learning (CBL) in small group teaching

**DOI:** 10.1038/s41598-025-99764-5

**Published:** 2025-05-04

**Authors:** Athanasios Hassoulas, Owen Crawford, Saiyonora Hemrom, Andreia de Almeida, Marcus J. Coffey, Megan Hodgson, Becky Leveridge, Diya Karwa, Alice Lethbridge, Huw Williams, Alex Voisey, Karen Reed, Sarju Patel, Keith Hart, Hannah Shaw

**Affiliations:** School of Medicine, University Hospital Wales, Cardiff University, Heath Park, 2nd Floor F-24, Cardiff, CF14 4XW UK

**Keywords:** Nervous system, Neurophysiology

## Abstract

The recent paradigm shift in teaching provision within higher education, following the COVID-19 pandemic, has led to blended models of learning prevailing in the pedagogic literature and in education practice. This shift has also resulted in an abundance of tools and technologies coming to market. Whilst the value of integrating technology into teaching and assessment has been well-established in the literature, the magnitude of choice available to educators and to students can be overwhelming. The current pilot investigated the feasibility of integrating key technologies in delivering technology-enhanced learning (TEL) case-based learning (CBL) within a sample of year two medical students. The cohort was selected at random, as was the control group receiving conventional CBL. Both groups were matched on prior academic performance. The TEL-CBL group received (1) in-person tutorials delivered within an immersive learning suite, (2) access to 3D anatomy software to explore during their self-directed learning time, (3) virtual reality (VR) guided anatomy exploration during tutorials, (4) access to a generative AI-based simulated virtual patient repository to practice key skills such as communication and history taking, and (5) an immersive medical emergency simulation. Metrics assessed included formative academic performance, student learning experience, and confidence in relation to communication and clinical skills. The results revealed that the TEL-CBL group outperformed their peers in successive formative assessments (*p* < 0.05), engaged thoroughly with the technologies at their disposal, and reported that these technologies enhanced their learning experience. Furthermore, students reported that access to the GenAI-simulated virtual patient platform and the immersive medical emergency simulation improved their clinical confidence and gave them a useful insight into what they can expect during the clinical phase of their medical education. The results are discussed in relation to the advantages that key emerging technologies may play in enhancing student performance, experience and confidence.

## Introduction

Recent watershed moments, such as the abrupt changes to teaching provision during the COVID-19 pandemic and the introduction of OpenAI’s ChatGPT platform, have served as important catalysts in the deeper integration of technology in teaching delivery and assessment. Whilst the higher education (HE) sector has been embracing various forms of technology-enhanced learning for quite some time, it takes disruptive events at a global scale to accelerate the rate of progress in a specific trajectory. Within just a few years of the COVID-19 pandemic, the mainstream introduction of Generative Artificial Intelligence (GenAI) has caused significant disruption within HE institutions^1^. The widescale applications and implications of artificial intelligence (AI) in education, as well as the implications of GenAI being integrated into curricula and assessment strategies, produces a new challenge in future-proofing teaching and assessment. Furthermore, students expect universities to deliver teaching that aligns with the way in which today’s younger generations consume and use information. The integration of new and emerging technologies in the workplace also emphasises the need for universities to prepare students adequately for these rapidly evolving working environments.

The need to weave new and emerging technologies into our teaching provision does not only relate to advances being made in GenAI, but equally to advances in relation to other technologies such as virtual reality (VR), augmented reality (AR), and immersive learning. These new and emerging technologies have the potential to enhance the student learning experience but present those in HE with the mammoth task of having to rapidly upskill staff in the use of these new tools and platforms, as well as considering how to appropriately integrate these new forms of teaching delivery into existing curricula. In no uncertain terms, these new technologies represent a fundamental constructive realignment of teaching activities with intended learning outcomes and assessments^2^. Crucial to any integration is an evaluation of the efficacy of technology-enhanced learning in HE.

As a starting point, most universities and departments are attempting to define what it is exactly that we mean by technology-enhanced learning (TEL). Kirkwood and Price (2014) for instance revealed in their review that the literature that the two key domains of TEL include (a) a change in the means through which teaching is delivered, and (b) a change in the way in which educators teach and learn, with the aim of transform learning and thereby enhancing the student experience^3^. The shift to blended learning over the past few years, especially during the post-pandemic era, has seen the introduction of various models that aim to integrate a blend of technologies alongside more traditional modes of teaching provision, as well as a blend of synchronous and asynchronous teaching activities^4^.One such example is the introduction of the Personalised, Evidence-Based, Inclusive Learning (PEBIL) model introduced by Hassoulas et al. (2023a), which has seen the integration of immersive technologies and digital tools in both distance as well as in-person programmes of study^5^. Case-based and problem-based curricula also lend themselves to such innovative blends on teaching delivery^6^.

The use of tools that enable greater interactivity with learning content has proven profoundly popular with undergraduate as well as postgraduate students^7^.The gamification of learning is one such area that has proven effective in engaging students as active participants in their learning as well as aligning teaching provision with experiences today’s students have outside the classroom, laboratory and lecture theatre. Immersive spaces and VR have proven to be a great form of entertainment as well as of learning^8^. Technologies such as VR/AR, immersive clinical scenarios, and genAI-simulated virtual patients provide medical students with a safe environment in which to apply their knowledge and practice key clinical, as well as even communication, skills^9,10^. As such, the application of these new technologies is not currently proposed as a replacement to any existing modes of teaching delivery, but rather as a means of enhancing teaching using technologies that are additional tools in our students’ toolboxes. Given the current state of the technologies at our disposal, TEL should aim to integrate tried-and-tested new technologies into curricula by carefully considering where learning and the student experience could indeed be enhanced. To this end, the Medical Schools Council in the United Kingdom, tasked with overseeing medical education provision across accredited British medical schools, has established a Digital Education Sub-group that includes representatives from all UK medical schools in considering the implementation of TEL in undergraduate curricula. Similarly, the International Association of Medical Educators has established the TEL Sub-committee that monitors trends and drivers within the sector, and the implications for medical education.

The current study introduced a pilot whereby TEL was applied in a small group teaching setting within the MBBCh undergraduate medical curriculum for a group of year two medical students during the spring semester (i.e., second semester of the academic year). The pilot incorporated the integration of immersive learning spaces (physical and virtual), VR, and a GenAI-simulated virtual patient platform. Previous studies have identified the growing need for, and relevance of, adaptive learning technologies^11^. Specifically, the use of emerging technologies has been identified to enhance the efficacy of learning^12^, as well the student experience^6,13^. Recent evidence has also highlighted the potential benefits of integrating VR into teaching provision as a means of enhancing student engagement^14^, and the importance of integrating GenAI platforms into curricula and assessment strategies as a means of preparing students adequately for the ethical and responsible use of such tools^15^. To the authors’ knowledge, there has not been a TEL-based study within an undergraduate medical curriculum that integrates such a broad range of key new technologies as a means of enhancing learning efficacy and students’ experience of learning. The aim of the current pilot was therefore to investigate whether the integration of these TEL-based approaches (a) enhanced performance on formative assessments, (b) enhanced the student learning experience, and (c) enhanced confidence in students’ communication and clinical skills.

## Methods

### Participants

The study consisted of an experimental group (n = 10) of year two undergraduate medical students, who participated in the technology-based (TEL) case-based learning (CBL) pilot that ran over the spring semester (January to May), and a control CBL group (n = 10) of year two undergraduate medical students who received conventional CBL over the same period. Ethical approval was sought and provided by the Cardiff University School of Medicine Research Ethics Committee (SoMREC). All protocols were approved, the pilot was conducted in accordance with the school’s guidelines and regulations, and consent was provided by participants. Both groups were matched in relation to key criteria that included: year 1 academic performance, year 2 progress test performance, number of graduate entry students in each group, age, and years of experience of the group facilitator/tutor. Table 1 below contains for more information about the TEL CBL group and the control CBL group:

**Table 1:** Confounder adjustment and matched characteristics of each group.

Matching criterion	TEL CBL group	Control CBL group
Average group age (years)	20.9	21.3
Gender ratio (male:female)	3:7	2:8
Graduate Entry Students	1	1
Year 1 average (%)	61.2	61.5
*Year 2 Progress tests (%)	42.9	43
Facilitator experience (years)	10	10

*The progress tests are year 5 single-based answer (SBA) knowledge papers administered to all medical students at Cardiff University between years 2 and 5 on the MBBCh programme, as a means of measuring trends in performance between this period.

### Apparatus and procedure

Both groups of students were assigned identical teaching sessions for each unit of study throughout the course of the spring semester. Specifically, each group attended the same lectures, practical tutorials, clinical skills sessions, community clinical learning, and small group teaching sessions. Each group was assigned an experienced CBL tutor, with both tutors being senior members of staff who have been CBL tutors since the CBL-based curriculum was introduced at Cardiff University’s School of Medicine eleven years ago. Both tutors have a research background in clinical science, are members of the undergraduate medicine curriculum team, and alongside small group tutorials also deliver lectures and units of study throughout the pre-clinical phase of the programme. The two tutors facilitated their respective groups independently and as per the study protocol. The tutor assigned to the conventional CBL group adhered to the curriculum’s well-established small group learning practices (i.e., no difference in delivery, lesson plan and structure of CBL). Whilst the TEL CBL tutor covered the identical material and adhered to the same CBL structure (e.g., 3 small group sessions per case, with two scenarios for students to work through), the lesson plan and delivery of the sessions differed by integrating the use of new and emerging technologies. This included access to a 5.5 × 5.5 m immersive learning suite, 3D anatomy software, asynchronous e-learning and virtual patient cases, a GenAI simulated virtual patient platform, and VR headsets equipped with immersive anatomy software. Both groups were allocated the same amount of time per CBL session, with the conventional CBL group exploring case content during group discussion, making use of the whiteboards to recap prior knowledge and to brainstorm. The TEL CBL group integrated the use of the technological tools at their disposal to recap prior knowledge, brainstorm, and to explore case content in further detail.

The immersive learning suite consists of projectors and sensors that transform the walls surrounding the space into an immersive environment whereby images and/or 360° videos can be projected onto the walls. The sensors also enable students to interact with contact projected onto the walls through the ‘touch’ feature. The suite was used to create immersive 3D anatomy sessions that were introduced to enhance students’ learning, with peer-teaching and teamwork activities (e.g., group quizzes) included in the lesson plan. The suite was also used to transform the space into a move ambulance so that students could practice key clinical skills in an immersive environment during an emergency simulation.

**Fig. 1:**
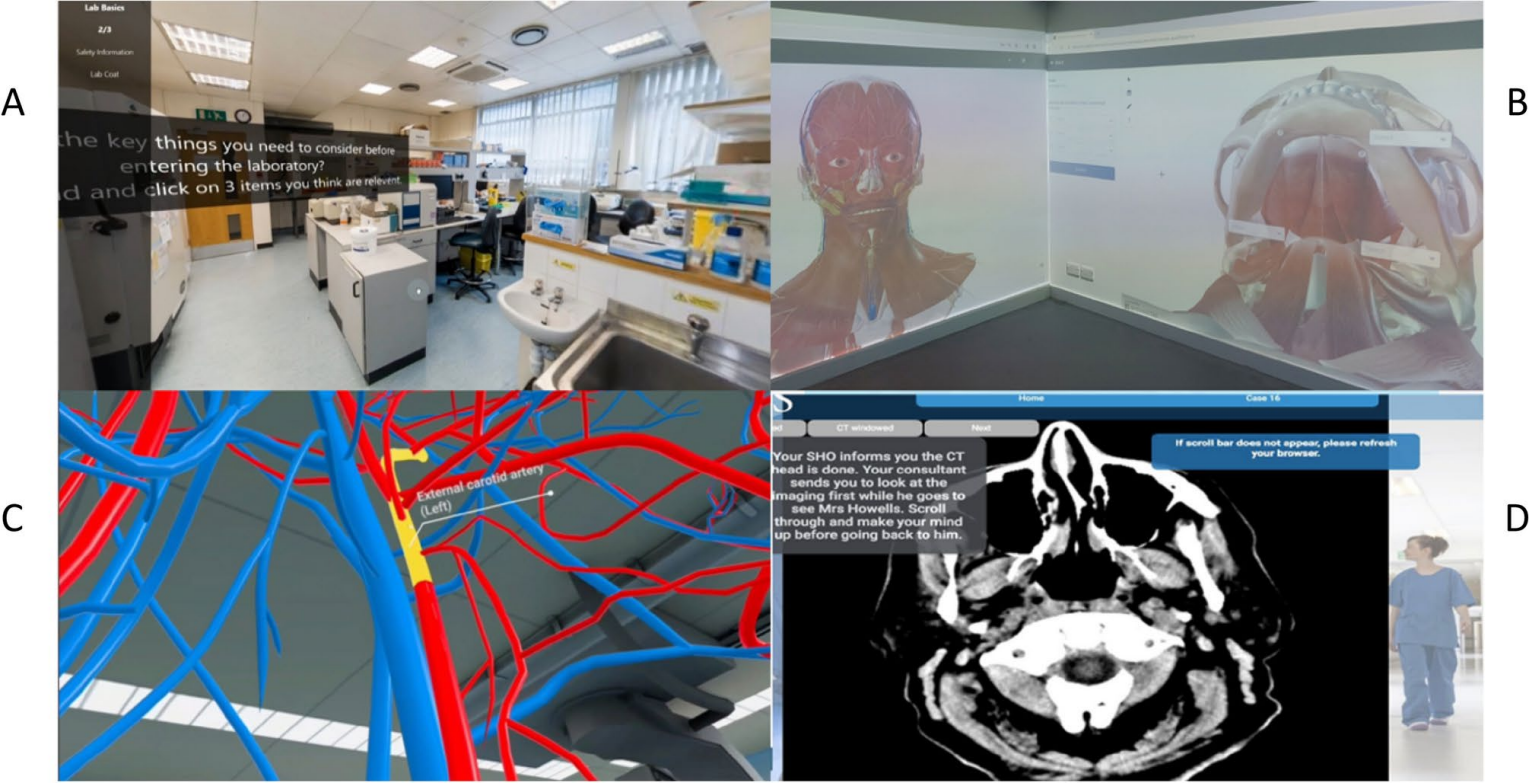
A sample of tools, technologies and facilities that were embedded within the TEL CBL pilot. These included (**A**) immersive virtual environments that could be explored on students’ individual devices, (**B**) a physical immersive learning suite that could accommodate the group of 10 TEL CBL students, (**C**) 3D anatomy software that was accessible using VR headsets as well (**D**) and interactive virtual patient clinical scenarios.

The 3D anatomy software used in the immersive learning suite, as well as embedded within asynchronous e-learning resources available to all year two students, was BioDigital Human (n.d.)^16^ and Complete Anatomy (n.d.)^17^. The pilot ChatGPT-based AI platform containing virtual patient scenarios was provided by SimPat (n.d.)^18^, who are a group of medical students developing generative AI (GenA1)-based virtual patient simulations that enable medical students to practice history taking and communication skills in their spare time. There were 12 Meta Quest 2 128GB VR headsets available for the 10 TEL CBL students, each containing the Virtual Medicine software (n.d.)^19^ that enabled students to explore human anatomy and physiology in a virtual and immersive learning environment. A snapshot of a few of the immersive resources and facilities used during this pilot are presented in Fig. 1.

During the spring semester, the year two students considered four units of study that explored various areas of clinical science and medicine. This included cardiology, neurology, orthopaedics, and gastrointestinal (upper) medicine. A blend of teaching activities was delivered during each unit, which included three small group teaching sessions. The TEL CBL group had access to the aforementioned technological tools and platforms during each of these three small group sessions. The use of the technology and platforms was included as a means of enhancing learning during these sessions, as opposed to replacing any element of the sessions specifically. Each small group session was facilitated by an experienced tutor, with an equally experienced tutor facilitating the control CBL group’s small group sessions. During the third and final session during each unit, a 20-item quiz was administered to both groups as a means of assessing knowledge acquisition and retention during the unit. The quizzes (4 in total, each consisting of 20 multiple-choice items) contained questions that aligned to the teaching activities that both groups of students engaged in (i.e., lectures, practical tutorial, community and clinical learning), with an emphasis on basic science questions (anatomy, physiology and histology where relevant). Students were also administered a survey that included Likert scale items, as well as open-ended questions in relation to the learning experience during the unit and confidence in their scientific knowledge and clinical competence. Specifically, students were asked to rate their experience of using the various technologies, tools and platforms, as well as to rate the extent to which these enhanced their learning and confidence. Engagement with the tools, platforms, devices and e-learning resources was also monitored by the group tutor.

### Data analysis

Quiz performance and engagement was analysed using an independent samples t-test in IBM SPSS (version 27). Student experience and confidence as reported by the TEL CBL group were captured in the form of quantitative descriptive statistics, with responses to the open-ended items being explored using content analysis, which has been reported as a well-suited approach to analysing qualitative health education data^2,20^.

## Results

Formative quiz data was collated during the final session of each unit for both the TEL CBL and control CBL groups. Engagement data was captured using the virtual learning environment, Blackboard, which provided detailed accounts of when and how often students were accessing the virtual patient cases and 3D e-learning resources. Data on the student learning experience and confidence in academic and clinical competency were captured at the end of the fourth unit.

### Quantitative analysis

Students assigned to the TEL CBL and control CBL groups demonstrated no difference in prior academic performance on the summative science papers nor the progress tests (ps > 0.05), illustrating that both groups were matched in relation to academic performance and ability. Table 2 below provides an overview of the formative quiz performance, post TEL intervention, for both the TEL CBL and Control CBL groups.

**Table 2:** Descriptive performance data for both groups of students.

	Number of students	Overall quiz mean	Standard deviation
TEL CBL group (%)	10	57.96	9.58
Control CBL Group (%)	10	49.75	11.48

**Fig. 2:**
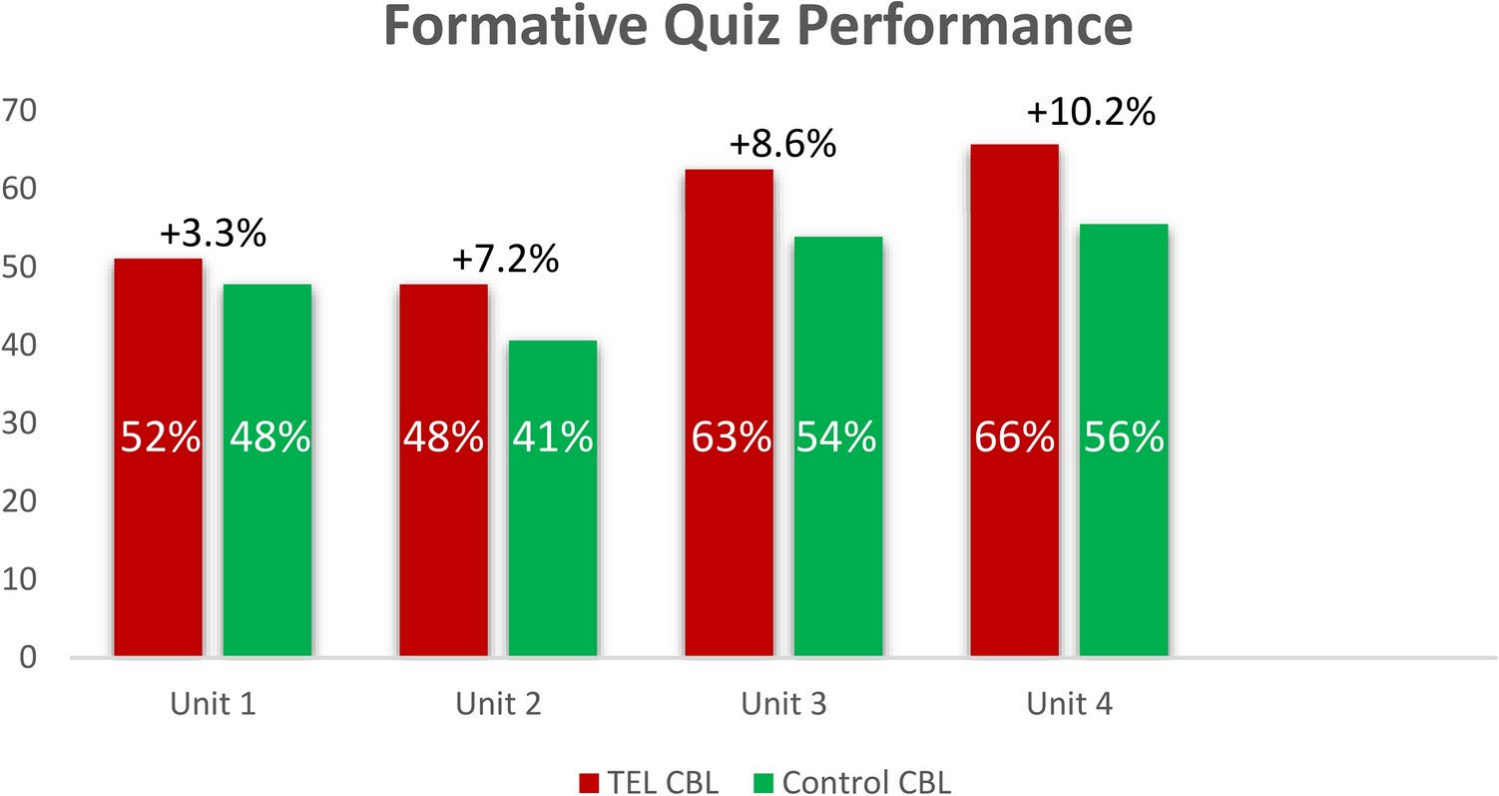
Quiz score difference per unit between the TEL CBL and Control CBL groups.

An independent-sample t-test was performed to compare quiz performance in the TEL CBL and Control CBL groups. This revealed a significant difference between the two groups, t(18) = 1.74, *p* < 0.05, on the overall scores across the four unit quizzes. The 95% confidence interval for the difference in means was 1.72 to 18.14. Whilst the TEL CBL group outperformed the control CBL group on each quiz, there was no statistically significant difference per quiz, but the difference was approaching significance during the latter quizzes. This trend is illustrated in Fig. 2 below, with the difference between the two groups growing larger with each consecutive quiz, demonstrating a cumulative effect.

**Fig. 3:**
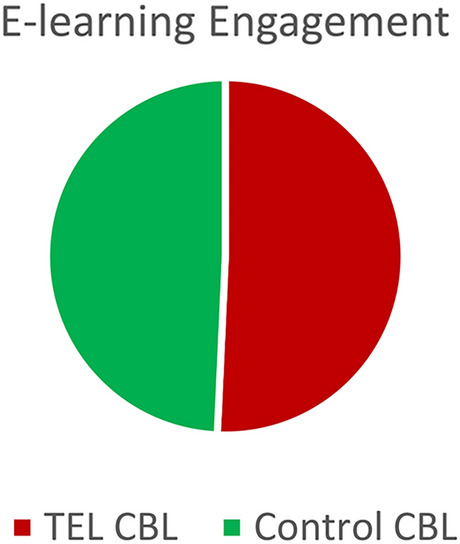
Engagement with interactive e-learning resources available to both groups.

When analysing engagement data relating to the e-learning resources and interactive asynchronous activities made available to both groups of students, there was no significant difference in engagement between the groups (*p* > 0.05), as further illustrate in Fig. 3. There was, however, a significant effect of e-learning usage found on academic performance, X^2^ = (1, n = 20) = 9.89, *p* < 0.05. This was revealed across both groups, using a median split to divide the TEL CBL and Control CBL groups into high and low quiz scorers, as well as using a median split for e-learning usage. This demonstrates that those who engaged more with the e-learning resources, which were made available to all students, performed better than those who engaged less.

**Fig. 4:**
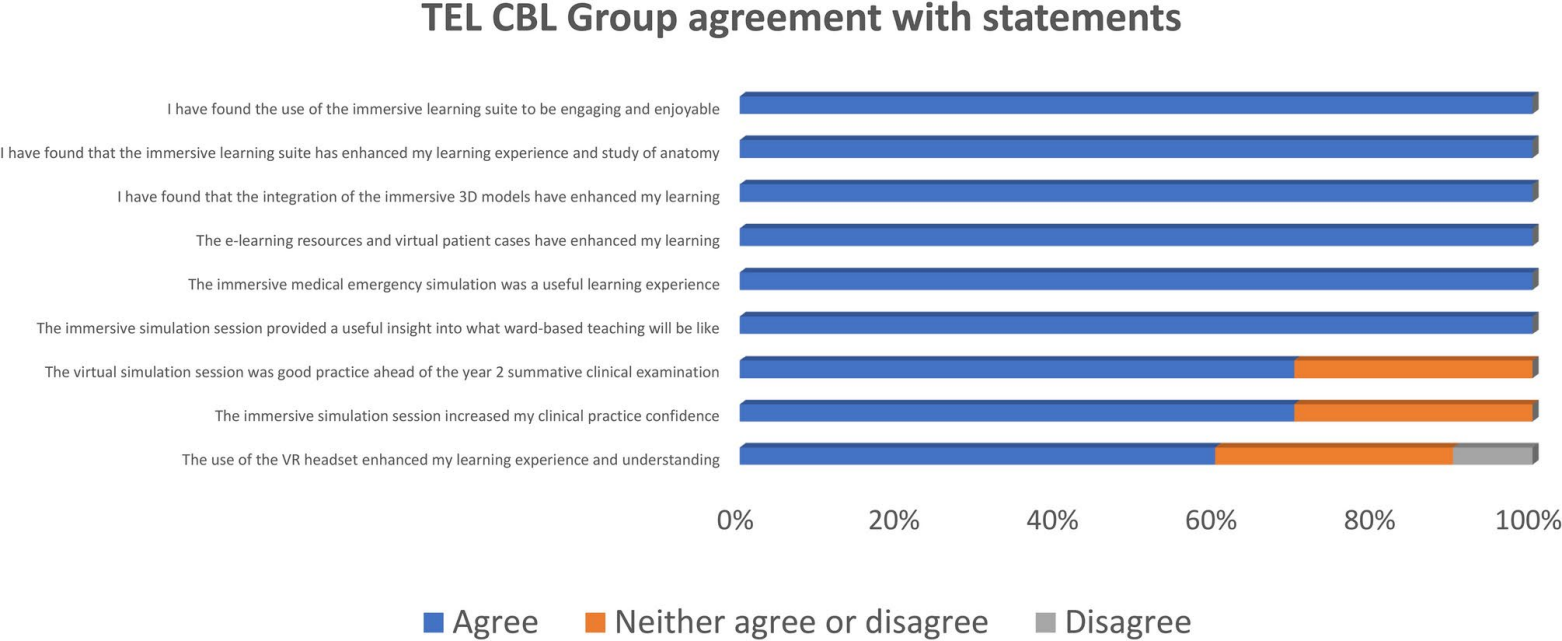
TEL CBL group student feedback on experience of new facilities and technologies.

Student experience of the technology-enhanced sessions was captured using a survey administered to the TEL CBL group. Responses highlighted a consensus among all students in the group that the immersive learning suite, 3D anatomy software, e-learning resources, and GenAI simulated virtual patient cases enhanced their experience and learning. Furthermore, use of the immersive suite proved a beneficial exercise in providing students with a glimpse into what ward-based teaching would be like in the years to come. Overall, 70% of students in the TEL CBL group reported that the use of these new facilities and technologies increased their confidence and proved to be good practice in preparation of their summative clinical examination at the end of the academic year. An area that has been identified as requiring further development is the use of the VR headsets in exploring anatomy and physiology, with 60% reporting that it was a valuable exercise whilst 20% were neutral and 10% disagreed. Figure 4 provides an overview of the student feedback collated.

### Qualitative analysis

The survey administered to students after the units of study also included open-ended items that were included as a means of capturing more granular feedback on the student experience. Content analysis was performed on the data using word frequency counts to explore the re-occurrence of key terms in the feedback provided by students in the TEL CBL group (inter-rater agreement of of 89%). This revealed that students consistently reported the benefits of being able to visualise anatomical structures in the immersive learning suite as well as through the use of the 3D anatomy software, with a few students also reporting that the use of the VR headsets were useful in that regard as well. Students also reported to find the use of the simulated virtual patient cases to be helpful in applying their learning to clinical scenarios and in practicing history taking and communication skills in a non-threatening environment. A key theme that emerged was the benefit of having access to a variety of tools, platforms and technologies as a means of enhancing and supplementing other teaching activities (see Table 3).

**Table 3:** Analysis and categorisation of qualitative feedback captured for the TEL CBL group.

What worked well? (listed in order of frequency reported)	Visualising anatomy in the immersive suite Being able to play around with 3D anatomy Using the VR headsets for anatomy Immersive quizzes Teamwork and immersive group activities Clinical application of science knowledge Virtual cases and practicing history taking Access to a variety of tools and tech Variety of approach to consolidate learning Good preparation for ISCE *(clinical exam)*
What could be improved upon? (listed in order of frequency reported)	More sessions available in the suite Streamline more with other teaching Be able to book the suite and other tools Make 3D anatomy available on own devices More immersive teamwork activities Use tools for other types of learning too

## Discussion

The current pilot explored the application of new technologies in an undergraduate case-based medical curriculum, specifically during the second year of the course. To the authors’ knowledge, this is the first study that has explored the use of immersive facilities (both physical and virtual), virtual reality, GenAI-simulated virtual patient cases, and interactive e-learning resources in enhancing the student experience and academic performance. Previous studies that have, explored the application of other technologies (such as platforms used for asynchronous resource creation, online live and pre-recorded teaching provision, group wikis and online logs) in CBL and PBL courses report that the integration of such tools and platforms has improved the student learning experience^21,22^.

Similarly to findings reported by Dunn and Kennedy (2019), who explored the integration of prior technological advances in enhancing academic performance, the current pilot revealed a significant difference in quiz scores between the TEL CBL group and the control CBL group^23^. Furthermore, the difference in group scores increased with each consecutive unit quiz. This demonstrates a cumulative effect over the course of the semester, corresponding with the greater exposure to the range of technological tools and platforms at the TEL CBL group’s disposal. A review by Tawafak et al. (2018) investigating trends in the literature regarding technology-enhanced learning and academic performance revealed a consensus that the integration of interactive technologies in teaching and learning improved performance by providing students with greater control over their learning, as well as guidance during their self-directed learning^24^. This relates also to an enhanced learning experience reported by these students, which aligns with the finding of the TEL CBL group in the current pilot study.

Students in the TEL CBL group overwhelmingly reported an enhanced learning experience through the integration of key new technologies and platforms in the small group sessions. Students unanimously agreed that the immersive learning suite helped consolidate learning, with the immersive quizzes providing an excellent group activity and team-building opportunities. Students in this group also all agreed that the use of the virtual patient cases and scenarios helped with the application of the basic science to clinical practice, leading to an increase in confidence in their abilities. Just over half the group, however, reported that the use of the VR headsets enhanced their learning and understanding. Possible reasons for this include the more solitary engagement with this sort of technology within a small group setting, which might not ‘fit’ as well with the specific lesson plan. Whilst all students found the ability to rotate, manipulate and dissect 3D anatomical models very useful (as also reported by Nakai et al., 2022)^25^, it is likely that disorientation and discomfort from wearing the headset over an extended period influenced students’ responses as well^26^. It is therefore crucial to capture further feedback from students who did not feel that the use of VR particularly enhanced their learning experience, and to consider ways in which key limitations in the application(s) of this technology can be addressed.

Students in the TEL CBL group also reported that the use of the physical immersive learning suite helped prepare them for the next phase of their medical education and training, with the immersive emergency simulations providing a safe space to fail and learn. They also reported that the use of the suite and virtual immersive clinical cases were useful in preparing for the clinical examination and improved their confidence in history taking and communication skills. This is consistent with findings by Taglieri et al. (2017), who reported that the use of virtual patient cases enhanced students’ clinical performance and confidence^27^. Mool et al. (2024) found that the use of generative AI-simulated patient cases enhanced the student learning experience, facilitating greater engagement than their control group (who were presented with multimedia patient case materials instead) in taking a detail patient history^28^. Whilst the TEL CBL group in the current pilot study reported the overall benefits of having access to a GenAI platform to practice history taking during self-directed learning time, they did also identify a few limitations of the technology. This included the rather circular nature of conversation the longer a student engaged with the AI-simulated patient. This is similar to limitations reported by Holderried et al. (2024), where their ChatGPT-based platform was reported to be a useful innovation but with a key limitation being the more socially desirable responses produced over time in certain instances that were not entirely medically plausible^29^. Whilst there is still work to be done in improving such novel platforms and AI technologies, it is worth considering the exponential growth in this area, with improved versions of existing platforms and new tools to be made available in the very near future.

The current pilot adjusted for confounders by ensuring that the TEL CBL and control CBL groups were matched in relation to prior academic performance and background, age of the students, and experience of each group’s tutor. As such, this increases confidence in any differences reported between the two groups being explained largely by the introduction of our intervention, namely the enhancing of teaching provision and exploration of core materials using the latest technological advances (as applicable to the higher education setting). Whilst the results illustrate the benefits of TEL small group teaching, it is important to note the small sample size and the importance of scaling up the study to explore whether the findings are replicable in a larger cohort of students. Despite the small sample size reported for this pilot, the findings are consistent with previous results reported by our group in relation to the student experience of using asynchronous and interactive e-resources to enhance learning^5^. Specifically, when sampling a group of 179 of our medical students, 66% reported a preference of asynchronous resources and a blended technology-focused approach to their learning. Furthermore, when our group previously evaluated a blended approach (which included asynchronous e-resources and virtual patient cases) for a unit of study, 94.7% (n = 226) reported that the integration of various technological tools enhanced their experience of the unit. With the ever-increasing variety in tools available to educators, identifying the most appropriate technologies for specific lesson plans, that are also pedagogically sound and inclusive, will be crucial moving forward.

## Data Availability

The raw data collated as part of this pilot, from both the TEL CBL and control CBL groups, has been uploaded to the Mendeley Data online repository. As this is a pilot study, with the current data forming an important part of a follow-up study that will shortly be underway, a link to the dataset will be made available upon reasonable request to the authors. Please contact Dr Athanasios Hassoulas (HassoulasA2@cardiff.ac.uk) for access to the dataset.
